# Supplementary material for: Modeling glioblastoma heterogeneity as a dynamic network of cell states
Source: Mol Syst Biol. 2021 Sep 16;17(9):e10105. doi: 10.15252/msb.202010105 (PMC8444284; doi:10.15252/msb.202010105)
Supplement: Supplementary file 5 — Source Data for Figure 3 [file MSB-17-e10105-s001.zip › Figure3A_sourcedata/GSEA_3065/hallmarks_state5.GseaPreranked.1623416302988/HALLMARK_ESTROGEN_RESPONSE_EARLY.html]

Details for gene set HALLMARK\_ESTROGEN\_RESPONSE\_EARLY[GSEA]

|  || Dataset | state5 |
| Phenotype | NoPhenotypeAvailable |
| Upregulated in class | na\_pos |
| GeneSet | HALLMARK\_ESTROGEN\_RESPONSE\_EARLY |
| Enrichment Score (ES) | 0.2839658 |
| Normalized Enrichment Score (NES) | 1.1529366 |
| Nominal p-value | 0.14814815 |
| FDR q-value | 0.26321244 |
| FWER p-Value | 0.885 |
Table: GSEA Results Summary

  

Fig 1: Enrichment plot: HALLMARK\_ESTROGEN\_RESPONSE\_EARLY      
 Profile of the Running ES Score & Positions of GeneSet Members on the Rank Ordered List

  

| PROBE | GENE SYMBOL | GENE\_TITLE | RANK IN GENE LIST | RANK METRIC SCORE | RUNNING ES | CORE ENRICHMENT || 1 | ABHD2 |  |  | 163 | 0.473 | 0.0156 | Yes |
| 2 | SLC16A1 |  |  | 186 | 0.449 | 0.0454 | Yes |
| 3 | SLC39A6 |  |  | 269 | 0.399 | 0.0648 | Yes |
| 4 | CD44 |  |  | 378 | 0.349 | 0.0777 | Yes |
| 5 | DHCR7 |  |  | 387 | 0.347 | 0.1017 | Yes |
| 6 | RETREG1 |  |  | 403 | 0.340 | 0.1244 | Yes |
| 7 | PODXL |  |  | 466 | 0.321 | 0.1405 | Yes |
| 8 | NAV2 |  |  | 478 | 0.318 | 0.1621 | Yes |
| 9 | IGF1R |  |  | 557 | 0.295 | 0.1745 | Yes |
| 10 | IL6ST |  |  | 627 | 0.279 | 0.1868 | Yes |
| 11 | GLA |  |  | 654 | 0.274 | 0.2036 | Yes |
| 12 | CELSR2 |  |  | 750 | 0.251 | 0.2109 | Yes |
| 13 | LRIG1 |  |  | 754 | 0.250 | 0.2285 | Yes |
| 14 | PRSS23 |  |  | 831 | 0.235 | 0.2368 | Yes |
| 15 | AMFR |  |  | 952 | 0.218 | 0.2389 | Yes |
| 16 | SLC26A2 |  |  | 1058 | 0.204 | 0.2417 | Yes |
| 17 | SLC2A1 |  |  | 1190 | 0.186 | 0.2404 | Yes |
| 18 | CELSR1 |  |  | 1257 | 0.178 | 0.2457 | Yes |
| 19 | ELOVL5 |  |  | 1320 | 0.169 | 0.2509 | Yes |
| 20 | GJA1 |  |  | 1399 | 0.162 | 0.2537 | Yes |
| 21 | FOS |  |  | 1412 | 0.161 | 0.2639 | Yes |
| 22 | ENDOD1 |  |  | 1552 | 0.147 | 0.2588 | Yes |
| 23 | XBP1 |  |  | 1564 | 0.145 | 0.2679 | Yes |
| 24 | ADCY9 |  |  | 1589 | 0.142 | 0.2754 | Yes |
| 25 | SEMA3B |  |  | 1604 | 0.141 | 0.2840 | Yes |
| 26 | FLNB |  |  | 2024 | 0.110 | 0.2447 | No |
| 27 | SLC1A4 |  |  | 2049 | 0.108 | 0.2498 | No |
| 28 | CXCL12 |  |  | 2165 | 0.101 | 0.2441 | No |
| 29 | CANT1 |  |  | 2299 | 0.092 | 0.2357 | No |
| 30 | HES1 |  |  | 2302 | 0.092 | 0.2420 | No |
| 31 | WFS1 |  |  | 2346 | 0.089 | 0.2436 | No |
| 32 | B4GALT1 |  |  | 2347 | 0.089 | 0.2499 | No |
| 33 | AFF1 |  |  | 2529 | 0.077 | 0.2351 | No |
| 34 | SLC7A5 |  |  | 2658 | 0.071 | 0.2258 | No |
| 35 | ABCA3 |  |  | 2662 | 0.070 | 0.2305 | No |
| 36 | AKAP1 |  |  | 2713 | 0.067 | 0.2297 | No |
| 37 | UGCG |  |  | 2764 | 0.065 | 0.2287 | No |
| 38 | SCARB1 |  |  | 2808 | 0.062 | 0.2283 | No |
| 39 | CA12 |  |  | 2885 | 0.058 | 0.2239 | No |
| 40 | AR |  |  | 2977 | 0.053 | 0.2175 | No |
| 41 | ABAT |  |  | 3020 | 0.051 | 0.2164 | No |
| 42 | WWC1 |  |  | 3113 | 0.046 | 0.2094 | No |
| 43 | ABLIM1 |  |  | 3183 | 0.042 | 0.2046 | No |
| 44 | MAST4 |  |  | 3192 | 0.041 | 0.2067 | No |
| 45 | FKBP5 |  |  | 3209 | 0.041 | 0.2078 | No |
| 46 | FASN |  |  | 3225 | 0.040 | 0.2090 | No |
| 47 | REEP1 |  |  | 3264 | 0.038 | 0.2074 | No |
| 48 | STC2 |  |  | 3306 | 0.036 | 0.2054 | No |
| 49 | KDM4B |  |  | 3364 | 0.033 | 0.2014 | No |
| 50 | MYBBP1A |  |  | 3380 | 0.032 | 0.2020 | No |
| 51 | MED13L |  |  | 3482 | 0.028 | 0.1926 | No |
| 52 | MREG |  |  | 3514 | 0.026 | 0.1910 | No |
| 53 | OLFM1 |  |  | 3559 | 0.025 | 0.1879 | No |
| 54 | MED24 |  |  | 3623 | 0.023 | 0.1824 | No |
| 55 | TIAM1 |  |  | 3782 | 0.016 | 0.1658 | No |
| 56 | SEC14L2 |  |  | 4023 | 0.006 | 0.1392 | No |
| 57 | MAPT |  |  | 4051 | 0.005 | 0.1365 | No |
| 58 | TPBG |  |  | 4075 | 0.004 | 0.1342 | No |
| 59 | FAM102A |  |  | 4131 | 0.002 | 0.1282 | No |
| 60 | TSKU |  |  | 4232 | -0.003 | 0.1171 | No |
| 61 | SYNGR1 |  |  | 4412 | -0.011 | 0.0977 | No |
| 62 | NADSYN1 |  |  | 4426 | -0.011 | 0.0970 | No |
| 63 | ISG20L2 |  |  | 4431 | -0.011 | 0.0974 | No |
| 64 | MYC |  |  | 4656 | -0.020 | 0.0736 | No |
| 65 | SVIL |  |  | 4953 | -0.031 | 0.0425 | No |
| 66 | FARP1 |  |  | 5041 | -0.034 | 0.0352 | No |
| 67 | TOB1 |  |  | 5047 | -0.035 | 0.0371 | No |
| 68 | SIAH2 |  |  | 5063 | -0.035 | 0.0380 | No |
| 69 | RPS6KA2 |  |  | 5067 | -0.035 | 0.0402 | No |
| 70 | RARA |  |  | 5104 | -0.037 | 0.0388 | No |
| 71 | NCOR2 |  |  | 5345 | -0.047 | 0.0151 | No |
| 72 | KLF10 |  |  | 5448 | -0.050 | 0.0072 | No |
| 73 | SNX24 |  |  | 5526 | -0.054 | 0.0024 | No |
| 74 | ELF1 |  |  | 5530 | -0.054 | 0.0060 | No |
| 75 | UNC119 |  |  | 5600 | -0.057 | 0.0023 | No |
| 76 | PDLIM3 |  |  | 5938 | -0.071 | -0.0305 | No |
| 77 | NRIP1 |  |  | 5948 | -0.072 | -0.0263 | No |
| 78 | BHLHE40 |  |  | 5950 | -0.072 | -0.0213 | No |
| 79 | ADD3 |  |  | 5984 | -0.073 | -0.0198 | No |
| 80 | TFAP2C |  |  | 6012 | -0.075 | -0.0175 | No |
| 81 | RHOBTB3 |  |  | 6115 | -0.081 | -0.0231 | No |
| 82 | FHL2 |  |  | 6169 | -0.083 | -0.0231 | No |
| 83 | SLC9A3R1 |  |  | 6276 | -0.089 | -0.0287 | No |
| 84 | ITPK1 |  |  | 6350 | -0.093 | -0.0303 | No |
| 85 | PPIF |  |  | 6616 | -0.109 | -0.0523 | No |
| 86 | INPP5F |  |  | 6724 | -0.115 | -0.0560 | No |
| 87 | TIPARP |  |  | 6731 | -0.116 | -0.0484 | No |
| 88 | PMAIP1 |  |  | 6872 | -0.128 | -0.0550 | No |
| 89 | CHPT1 |  |  | 6977 | -0.135 | -0.0570 | No |
| 90 | SH3BP5 |  |  | 7076 | -0.143 | -0.0578 | No |
| 91 | RBBP8 |  |  | 7099 | -0.145 | -0.0498 | No |
| 92 | FKBP4 |  |  | 7326 | -0.170 | -0.0630 | No |
| 93 | NBL1 |  |  | 7402 | -0.181 | -0.0585 | No |
| 94 | BAG1 |  |  | 7474 | -0.189 | -0.0529 | No |
| 95 | MYOF |  |  | 7523 | -0.194 | -0.0444 | No |
| 96 | NXT1 |  |  | 7688 | -0.217 | -0.0473 | No |
| 97 | FDFT1 |  |  | 7852 | -0.244 | -0.0482 | No |
| 98 | BLVRB |  |  | 8154 | -0.309 | -0.0598 | No |
| 99 | TUBB2B |  |  | 8224 | -0.332 | -0.0438 | No |
| 100 | DYNLT3 |  |  | 8282 | -0.349 | -0.0252 | No |
| 101 | CCND1 |  |  | 8346 | -0.368 | -0.0058 | No |
| 102 | ARL3 |  |  | 8412 | -0.391 | 0.0149 | No |
| 103 | RAB31 |  |  | 8831 | -0.687 | 0.0172 | No |
Table: GSEA details [plain text format]

  

Fig 2: HALLMARK\_ESTROGEN\_RESPONSE\_EARLY: Random ES distribution      
 Gene set null distribution of ES for **HALLMARK\_ESTROGEN\_RESPONSE\_EARLY**
